# Aberrant Amplitude of Low-Frequency Fluctuations in Different Frequency Bands in Patients With Parkinson’s Disease

**DOI:** 10.3389/fnagi.2020.576682

**Published:** 2020-12-03

**Authors:** Zhaoxiu Wang, Yanjun Liu, Xiuhang Ruan, Yuting Li, E. Li, Guoqin Zhang, Mengyan Li, Xinhua Wei

**Affiliations:** ^1^Department of Radiology, Guangzhou First People’s Hospital, School of Medicine, South China University of Technology, Guangzhou, China; ^2^Institute of Biomedical and Health Engineering, Shenzhen Institutes of Advanced Technology, Chinese Academy of Sciences, Shenzhen, China; ^3^Department of Radiology, Guangzhou First People’s Hospital, Guangzhou Medical University, Guangzhou, China; ^4^Department of Neurology, Guangzhou First People’s Hospital, School of Medicine, South China University of Technology, Guangzhou, China; ^5^Padova Neuroscience Center (PNC), University of Padova, Padua, Italy

**Keywords:** resting-state functional MRI, Parkinson’s disease, amplitude of low-frequency fluctuation, frequency band, spontaneous brain activity

## Abstract

Previous studies reported abnormal spontaneous neural activity in Parkinson’s disease (PD) patients using resting-state functional magnetic resonance imaging (R-fMRI). However, the frequency-dependent neural activity in PD is largely unknown. Here, 35 PD patients and 35 age- and education-matched healthy controls (HCs) underwent R-fMRI scanning to investigate abnormal spontaneous neural activity of PD using the amplitude of low-frequency fluctuation (ALFF) approach within the conventional band (typical band: 0.01–0.08 Hz) and specific frequency bands (slow-5: 0.010–0.027 Hz and slow-4: 0.027–0.073 Hz). Compared with HCs, PD patients exhibited increased ALFF in the parieto-temporo-occipital regions, such as the bilateral inferior temporal gyrus/fusiform gyrus (ITG/FG) and left angular gyrus/posterior middle temporal gyrus (AG/pMTG), and displayed decreased ALFF in the left cerebellum, right precuneus, and left postcentral gyrus/supramarginal gyrus (PostC/SMG) in the typical band. PD patients showed greater increased ALFF in the left caudate/putamen, left anterior cingulate cortex/medial superior frontal gyrus (ACC/mSFG), left middle cingulate cortex (MCC), right ITG, and left hippocampus, along with greater decreased ALFF in the left pallidum in the slow-5 band, whereas greater increased ALFF in the left ITG/FG/hippocampus accompanied by greater decreased ALFF in the precentral gyrus/PostC was found in the slow-4 band (uncorrected). Additionally, the left caudate/putamen was positively correlated with levodopa equivalent daily dose (LEDD), Hoehn and Yahr (HY) stage, and disease duration. Our results suggest that PD is related to widespread abnormal brain activities and that the abnormalities of ALFF in PD are associated with specific frequency bands. Future studies should take frequency band effects into account when examining spontaneous neural activity in PD.

## Introduction

As one of the most common neurodegenerative disorders, Parkinson’s disease (PD) is characterized by motor symptoms, such as resting tremor, bradykinesia, rigidity, postural instability, and non-motor symptoms, including autonomic dysfunction, mood disorders, and cognitive impairments (Herrington et al., 2017). The main neuropathological characteristics of PD are the loss of dopamine neurons in the substantia nigra and the formation of intracellular Lewy inclusion bodies; however, these characteristics do not sufficiently explain the heterogeneity of symptoms and the progression of the disease (Tuovinen et al., 2018). To date, the underlying disease-related neural mechanism has not been completely understood.

Advanced neuroimaging techniques have been extensively employed to explore the neural substrates of neurologic and psychiatric diseases. Positron emission tomography (PET) or single photon emission computed tomography (SPECT) studies have shown that PD patients present abnormal cerebral metabolism in the basal ganglia and some cortical regions (Thobois et al., 2001; Albrecht et al., 2019). Resting-state functional magnetic resonance imaging (R-fMRI) reveals the phenomenon of spontaneous neuronal activity at rest by examining spontaneous fluctuations of the blood oxygen level-dependent (BOLD) signal, providing a reliable measure of baseline brain activity (Gusnard et al., 2001; Fox and Raichle, 2007; Liu et al., 2013). Using R-fMRI, Wu et al. (2011) and Tuovinen et al. (2018) reported PD patients existed abnormal connectivity in the cortico-striato-thalamic loop and cortico-basal-cerebellar connectome in PD patients. As a reliable measure of the magnitude of spontaneous BOLD signals, the amplitude of low-frequency fluctuations (ALFF) has been widely applied to assess spontaneous neural activity related to cerebral physiological and pathological states (Skidmore et al., 2013; Zhang et al., 2015; Wang et al., 2016). Tang et al. (2017) reported decreased ALFF in the bilateral lingual gyrus and left putamen in PD patients compared with healthy controls. Furthermore, a meta-analysis (Pan et al., 2017) reported that the most consistent and replicable findings in PD patients were decreased ALFF in the bilateral supplementary motor areas, left putamen, left premotor cortex, and left inferior parietal gyrus and increased ALFF in the right inferior parietal gyrus.

To date, most ALFF studies in PD have focused on the conventional low-frequency band of 0.01–0.08 Hz (Skidmore et al., 2013; Xiang et al., 2016) because the BOLD signal of this band is thought to reflect spontaneous brain activity (Fox and Raichle, 2007). However, studies have proposed that different frequencies of neural oscillations in the human brain may be sensitive to activity in different regions and can be applied to reflect distinct physiological functions of brain activity (Buzsaki and Draguhn, 2004; Zuo et al., 2010). The frequency spectrum was decomposed into five different frequency bands, including slow-6 (0–0.01 Hz), slow-5 (0.01–0.027 Hz), slow-4 (0.027–0.073 Hz), slow-3 (0.073–0.198 Hz), and slow-2 (0.198–0.25 Hz). Slow-6, slow-3, and slow-2 oscillations were discarded because they mainly reflect very low-frequency drift, white matter signals, and high-frequency physiological noise, whereas slow-4 and slow-5 oscillations primarily correlated with gray matter are beneficial to identify correlations of functional processing and diseases (Salvador et al., 2008; Zuo et al., 2010). Low-frequency oscillations at different frequency bands exhibit different properties and physiological functions (Zuo et al., 2010). Indeed, by examining the ALFF in the slow-4 and slow-5 bands, frequency-dependent brain changes were detected in schizophrenia (Yu et al., 2014), major depressive disorder (Wang et al., 2016), social anxiety disorder (Zhang et al., 2015), and Alzheimer’s disease (Liu et al., 2014). By performing an independent validation study, a meta-analysis (Wang et al., 2018) suggested that decreased ALFF in the putamen was the most consistent finding in PD patients and was mainly found in the subfrequency band of slow-4, but the only two original studies (Zhang et al., 2013; Hou et al., 2014) on these subfrequency analyses may have been performed on different medication statuses. These results require further research. The condition of dopaminergic medication was actually unclear in the study of Zhang et al. (2013), whereas another ALFF study of PD in the subfrequency band was definitely performed in the off-medication state (Hou et al., 2014). Dopaminergic drugs have been proven to profoundly modulate the power and coherence of low-frequency oscillations in cortico-striato-thalamic systems (Honey et al., 2003). To our knowledge, no research has investigated the frequency-dependent ALFF in an on-medication state in PD patients to date. It would be necessary to differentiate the frequency bands to examine the ALFF in PD patients in the on-medication state.

We hypothesized that the changes in ALFF in PD are associated with the frequency band. In the current study, we investigated the altered ALFF of PD patients in the on-medication state in the typical band (0.01–0.08 Hz) and further in specific frequency bands (slow-5: 0.01–0.027 Hz; slow-4: 0.027–0.073 Hz). Moreover, correlations between the abnormal ALFF values and clinical measures were also explored. We expected that the characteristics of spontaneous neural activity in PD will be discovered in a specific frequency band.

## Materials and Methods

### Subjects

In this study, we recruited 37 patients diagnosed with PD who were partly overlapped with our previous study focusing on global synchronization (Li et al., 2019) and 36 matched healthy controls (HCs), which was accordant with the normal control in this paper. PD patients were diagnosed according to the clinical criteria of the Movement Disorder Society (MDS) (Postuma et al., 2015). The exclusion criteria for PD patients were as follows: (i) diagnosis uncertain for PD or suspicious of parkinsonism syndrome (vascular, drug-induced, toxin-induced, and postinfectious parkinsonism); (ii) a history of brain surgery (thalamotomy and posteroventral pallidotomy, deep brain stimulation, and organ transplantation); (iii) severe cardiovascular disease, respiratory disease, or severe symptoms of dementia that fail to cooperate; (iv) a pacemaker embedded in the body, which is forbidden in MRI scan. All of the PD patients received a stable dose of levodopa medication treatment and were in the on-medication state during clinical assessments and MRI data collection. No drug-naïve patients were included in this study. The HC subjects were healthy with no history of neurological disease, no psychiatric illness, and no neuroanatomical abnormalities and were included as Mini-Mental State Exam (MMSE) (Folstein et al., 1975) ≥24. Both PD and HC subjects were enrolled from Guangzhou First People’s Hospital. This research was approved by the Institutional Review Board (IRB) of Guangzhou First People’s Hospital, and all participants provided written informed consent before the scan.

### Clinical Assessments

Clinical assessments (including motor and non-motor symptoms) were measured across all PD patients. The severity of motor symptoms was recorded using the motor part (part III) of the Unified Parkinson’s Disease Rating Scale (UPDRS-III) (Goetz et al., 2008), and the severity of PD was evaluated by the Hoehn and Yahr (HY) scale (Hoehn and Yahr, 1998). Higher UPDRS-III scores indicated decreased movement ability. All participants underwent MMSE evaluations to measure general cognitive abilities. The levodopa equivalent daily dose (LEDD) was also collected from all PD patients.

### Data Acquisition

All imaging data were obtained using a 3-T MRI scanner (Magnetom Verio, Siemens Healthcare, Erlangen, Germany). Head movement was minimized by using foam padding, and scanner noise was diminished by earplugs. All of the subjects were required to lie quietly and remain awake with their eyes closed during the whole scanning process. The functional images were obtained using an echo-planar imaging sequence with the following parameters: repetition time (TR) = 2,000 ms, echo time (TE) = 21 ms, flip angle (FA) = 78, matrix size = 64 × 64, slice thickness = 4 mm, pixel spacing = 3.5 mm × 3.5 mm, and field of view (FOV) = 224 mm × 224 mm. After the functional scan, T1-weighted images were acquired with the following parameters: TR = 1,900 ms, TE = 2.22 ms, matrix = 256 × 215, FA = 9, pixel spacing = 0.488 × 0.488, and slice thickness = 1 mm.

### Data Preprocessing

All imaging data were preprocessed and analyzed using the toolkits of DPABI^1^ and Statistical Parametric Mapping (SPM12)^2^ on a MATLAB platform. Preprocessing procedures included the following: (i) removal of the first 10 volumes from 220 volumes to allow the subjects to adapt to the circumstances; (ii) slice timing correction for acquisition delay between slices; (iii) head-motion correction (excessive motion was defined as translation or rotation of >2.5 or 2.5°mm); (iv) regression of nuisance covariates including linear trend, white matter signals, cerebral spinal fluid signal, and Friston-24 parameters of head motions; (v) spatial normalization into the Montreal Neurological Institute (MNI) space by DARTEL (Ashburner, 2007) with a resampled voxel size of 3 × 3 × 3 mm^3^; (vi) before smoothing with a 6-mm full width at half maximum Gaussian kernel, data were separately filtered using the typical band (0.01–0.08 Hz), slow-5 band (0.01–0.027 Hz), and slow-4 band (0.027–0.073 Hz).

### ALFF Analysis

All voxels were converted from the time domain to the frequency domain using a fast Fourier transform to obtain the power spectrum. Then, the average square root of the power spectrum for each voxel was computed and regarded as ALFF (Zang et al., 2007). For standardization, all ALFF maps were converted into z-maps by subtracting the global mean value and then dividing by the standard deviation. The subsequent statistical and correlative analyses were based on the standardized ALFF maps.

### Statistical Analysis

To obtain demographic and clinical characteristics, group differences between the PD and HC groups in age, mean framewise displacement (FD) (Jenkinson et al., 2002), education year, and MMSE scores were analyzed using a two-sample *t*-test, and *p* < 0.05 was considered statistically significant.

To explore the ALFF differences between two groups, firstly, a two-sample *t*-test was performed on ALFF maps of a typical band (0.01–0.08 Hz) within gray matter mask (threshold at 20%), with age, gender, and head motions (mean FD) as covariates. Then, mixed effect analysis was performed on two groups and their ALFF of slow-5 and slow-4, by applying two-way repeated-measure analysis of variance (ANOVA) to examine the effects of group and frequency band, with group (PD and HC) serving as a between-subject factor and frequency band (slow-4 and slow-5) as a repeated-measures factor. Furthermore, within the brain regions showing interactions of group and frequency band, *post hoc* two-sample *t*-tests were performed between PD and HC for slow-5 and slow-4, respectively. Additionally, on account of abnormal ALFF in the caudate/putamen widely reported in previous studies, ANOVA was also examined within the bilateral caudate and putamen, which were extracted from the automated anatomical label (AAL) template. All statistical maps were corrected by Gaussian random field (GRF) with voxel *p* < 0.005 (or *p* < 0.05) and cluster *p* < 0.05.

Brain regions exhibiting significant ALFF differences between the PD and HC were extracted as regions of interest (ROIs). To identify the relationships between the mean ALFF value of these regions and clinical measurements, the Pearson correlation analysis was applied separately, and all correlative results were corrected by the false discovery rate (FDR) with *p* < 0.05.

## Results

### Demographic and Clinical Data

According to the criteria of excessive head motion mentioned previously, the R-fMRI data from two patients and one control were excluded due to excessive head motion. Demographic and clinical details of the remaining 35 patients and 35 controls are presented in Table 1. No significant differences (*p* > 0.05) were observed between the PD and HC groups in age, years of education, and mean head motions (mean FD). As expected, significant group differences in MMSE scores (*p* = 0.0026) were noted. PD patients exhibited lower MMSE scores than did HC subjects.

**TABLE 1:** Demographic and clinical characteristics.

	PD (*n* = 35)	HC (*n* = 35)	Statistical *p*-value
Age (years old) (range)	63.31 ± 10.45 (35–90)	59.57 ± 5.94 (47–81)	0.0679
Gender (female/male)	21/14	24/11	NA
Education years	9.94 ± 3.50	11.08 ± 2.84	0.1388
Mean FD (mm)	0.08 ± 0.04	0.09 ± 0.06	0.3556
Disease duration (years)	4.54 ± 4.30	NA	NA
MMSE	25.31 ± 4.46	27.88 ± 2.10	0.0026
HY	2.34 ± 0.74	NA	NA
UPDRS-III	32.00 ± 15.84	NA	NA
LEDD (mg)	459.81 ± 389.56	NA	NA

*PD, patients with Parkinson’s disease; HC, healthy controls; FD, framewise displacement of Jenkinson; MMSE, Mini-Mental State Examination; HY, Hoehn and Yahr scale; UPDRS-III, part three (motor part) of Unified Parkinson’s Disease Rating Scale; LEDD, levodopa equivalent daily dose. Data are presented as mean ± standard deviation. The statistical *p*-value was obtained by two-sample *t*-tests with a significance level of *p* < 0.05.*

### ALFF Analysis

**FIGURE 1:**
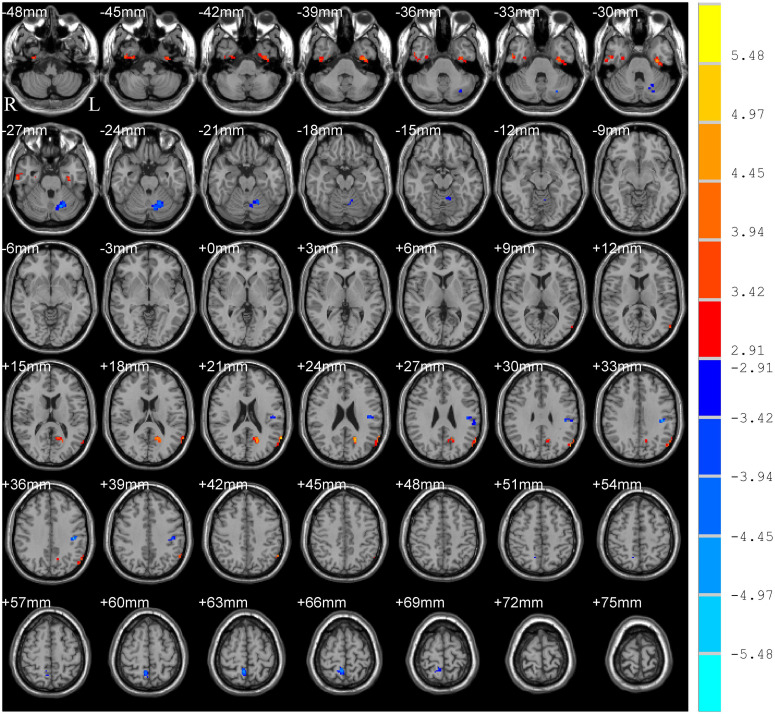
T-map of ALFF differences in the typical band (0.01–0.08 Hz). The results were obtained by two-sample *t*-test [GRF corrected, voxel *p* < 0.005, cluster *p* < 0.05, threshold of *t* > 2.91, and cluster size >46 voxels (1,242 mm^3^)]. The color bar on the right indicates statistical *t*-value. Warm (cold) overlays indicate higher (lower) ALFF in PD patients. L/R, left/right.

Significant group differences in typical bands are presented in Table 2 and Figure 1. Compared to HC subjects, the PD patients exhibited significantly increased ALFF in the right inferior temporal gyrus/fusiform gyrus/parahippocampus (ITG/FG/ParaHip), left FG/ITG, left angular gyrus/posterior middle temporal gyrus (AG/pMTG), and left cuneus/calcarine, accompanied by decreased ALFF in the left postcentral/supramarginal gyrus (PostC/SMG), right precuneus, and left cerebellum.

**TABLE 2:** Brain regions showing ALFF differences between groups in typical band and the main effect of group.

Brain regions	BA	Cluster size (voxels/mm^3^)	MNI coordinates (x y z)	Peak *t*-value
**Typical band (0.01–0.08 Hz)**				
R-ITG/FG/ParaHip	20/21/36	74/1,998	63 −9 −27	4.33
L-FG/ITG	20	80/2,160	−33 −12 −30	4.79
L-AG/Pmtg	39/21	60/1,620	−63 −57 21	5.24
L-Cuneus/calcarine	23/17	50/1,350	−18 −60 24	5.20
L-PostC/SMG	48/3	50/1,350	−42 −24 33	−5.11
R-Precuneus	5/7	51/1,377	6 −54 63	−4.89
L-Cerebellum_6	NA	88/2,376	−18 −63 −27	−4.68
**Main effect of group (slow-5 and slow-4)**				
R-FG/ParaHip	20/36	62/1,674	39 −21 −27	4.09
R-ITG	21/20	55/1,485	63 −9 −27	4.55
L-FG/ITG	20	66/1,782	−36 −9 −39	4.62
L-AG/pMTG	39/21	61/1,647	−63 −57 21	4.99
L-Cuneus/Calcarine	23/17	47/1,269	−18 −60 24	4.94
L-PostC/SMG	48/3	49/1,323	−42 −24 33	−5.46
R-Precuneus	5/7	50/1,350	6 −54 63	−5.03
L-Cerebellum_6	NA	81/2,187	−18 −63 −27	−4.72

*BA, Brodmann’s area; MNI, Montreal Neurological Institute; L/R, left/right; ITG, inferior temporal gyrus; FG, fusiform gyrus; ParaHip, parahippocampus gyrus; AG, angular gyrus; pMTG, posterior middle temporal gyrus; PostC, postcentral gyrus; SMG, supramarginal gyrus. The results in the typical band were obtained by two-sample *t*-tests, and the results of main group effects were obtained by two-way repeated-measures ANOVA [GRF corrected, voxel *p* < 0.005, cluster *p* < 0.05, threshold of *t* > 2.91, cluster size >46 voxels (1,242 mm^3^)]. Positive (negative) *t*-value indicates that PD patients had higher (lower) ALFF.*

**FIGURE 2:**
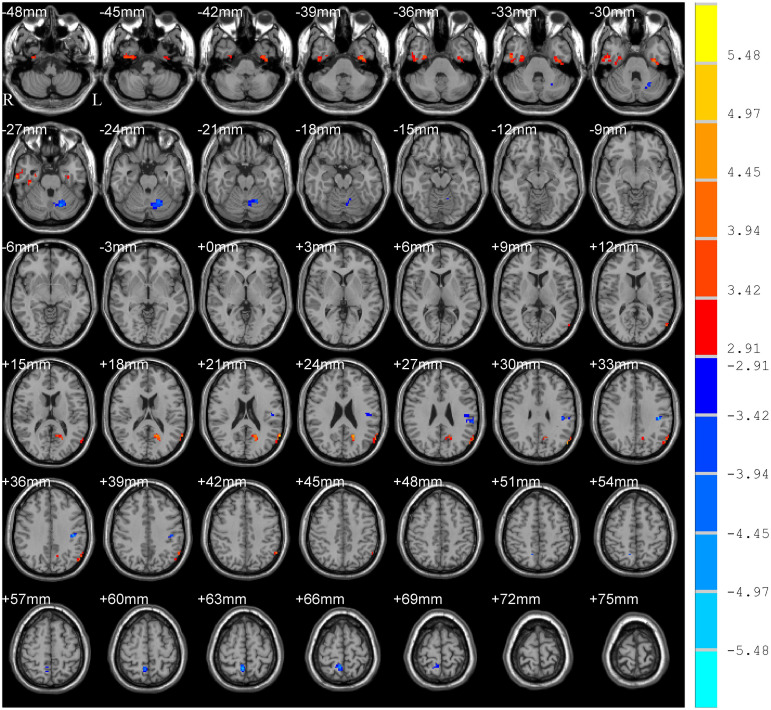
Main effect of group on ALFF. The results were obtained by two-way repeated-measures ANOVA [GRF corrected, voxel *p* < 0.005, cluster *p* < 0.05, threshold of *t* > 2.91, and cluster size >46 voxels (1,242 mm^3^)]. The color bar on the right indicates statistical *t*-value. Warm (cold) overlays indicate higher (lower) ALFF in PD patients. L/R, left/right.

**FIGURE 3:**
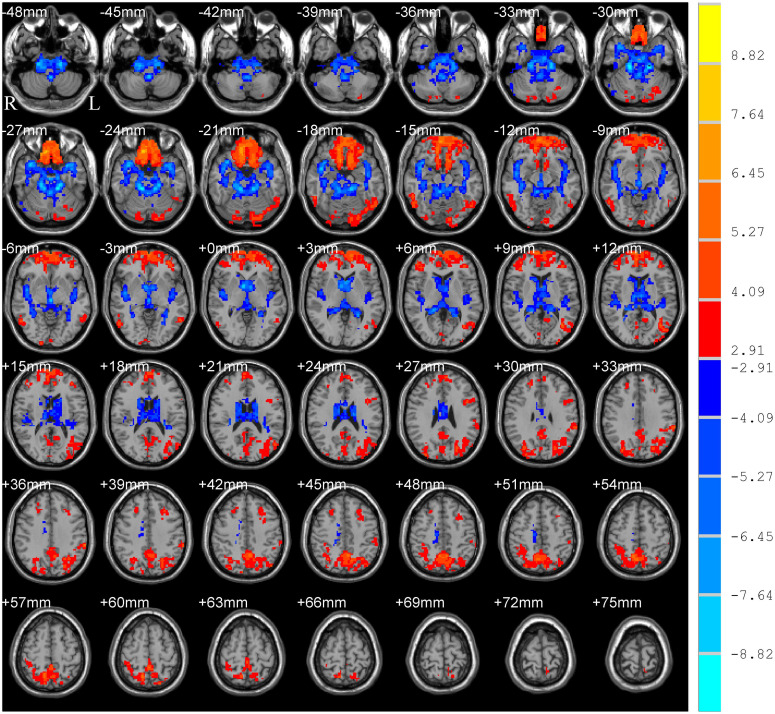
Main effect of frequency band on ALFF. The results were obtained by two-way repeated-measures ANOVA [GRF corrected, voxel *p* < 0.005, cluster *p* < 0.05, threshold of *t* > 2.91, and cluster size >39 voxels (1,053 mm^3^)]. The color bar on the right indicates statistical *t*-value. Warm (cold) overlays indicate higher (lower) ALFF in slow-5 band than in slow-4. L/R, left/right.

The main effect of group (PD and HC) is shown in Table 2 and Figure 2, which displayed accordant brain regions reported in typical bands that confirmed the group differences. The main effect of the frequency band (slow-5 and slow-4) is shown in Figure 3. Brain regions showing significant increased ALFF in the slow-5 band were identified in the bilateral middle prefrontal cortex, bilateral precuneus/posterior cingulate cortex, bilateral occipital/ITG/MTG, left SMG, and left posterior cerebellum, whereas increased ALFF in the slow-4 band was found in the bilateral superior temporal gyrus/Heschl gyrus, bilateral insula, bilateral caudate, bilateral hippocampus, and bilateral anterior cerebellum. It is obvious that greater ALFF in the slow-5 band was identified in widespread cortical regions, whereas greater ALFF in the slow-4 band was identified mainly in the temporal cortex and subcortical regions.

**FIGURE 4:**
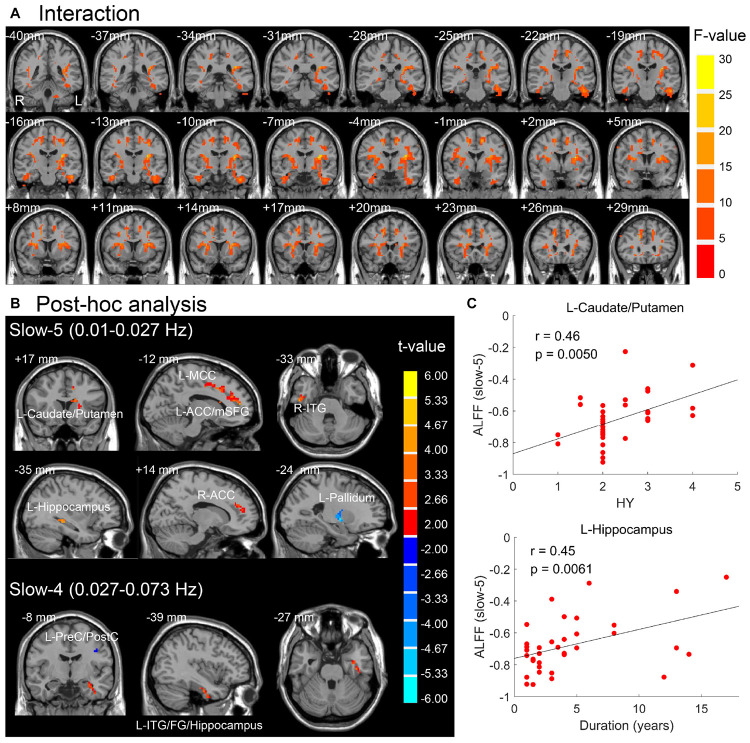
**(A)** Interaction effect between group and frequency band (*p* < 0.05, *F* > 3.98, uncorrected). **(B)** *Post hoc* two-sample *t*-test for slow-5 and slow-4 (voxel *p* < 0.05, threshold of *t* > 2.00, uncorrected). Warm (cold) overlays indicate higher (lower) ALFF in PD than in HC. **(C)** Significant correlations between mean ALFF of brain regions in panel **(B)** and clinical assessments (FDR corrected, *p* < 0.05). L/R, left/right; ACC/MCC, anterior/middle cingulate cortex; mSFG, medial superior frontal gyrus; ITG, inferior temporal gyrus; FG, fusiform gyrus; PreC/PostC, precentral/postcentral gyrus; HY, Hoehn and Yahr scale.

The brain regions resulted from interactions between frequency band and group on ALFF (*p* < 0.05, *F* > 3.98, uncorrected) are located in the basal ganglia, insula, ITG, FG, hippocampus gyrus, supplementary motor area, anterior and middle cingulate cortex, medial superior frontal gyrus, and cerebellum (Figure 4A). After *post hoc* analysis (voxel *p* < 0.05, threshold of *t* > 2.00, uncorrected), the brain regions demonstrating greater increased ALFF in PD patients in the slow-5 band were identified in the left caudate/putamen, left anterior cingulate cortex/medial superior frontal gyrus (ACC/mSFG), left middle cingulate cortex (MCC), right ITG, right ACC, and left hippocampus, along with greater decreased ALFF in left pallidum, whereas accompanied by greater decreased ALFF in the precentral gyrus/PostC, greater increased ALFF of PD was identified in left ITG/FG/hippocampus in the slow-4 band (Table 3 and Figure 4B). The abnormal left caudate/putamen in the slow-5 band was positively correlated with the HY stage, and the left hippocampus was positively correlated with disease duration as shown in Figure 4C and Table 4 (*p* < 0.05, FDR corrected).

**TABLE 3:** *Post hoc* analysis based on the interaction between group and frequency band.

Brain regions	BA	Cluster size (voxels/mm^3^)	MNI coordinates (x y z)	Peak *t*-value
**Slow-5 (0.01–0.027 Hz)**				
L-Caudate/Putamen	NA	28/756	−18 15 18	4.95
L-ACC/mSFG	32	37/999	−12 51 12	4.11
L-MCC	32	27/729	−12 18 36	3.95
R-ITG	20	27/729	51 −15 −33	4.16
R-ACC	32	23/621	15 42 21	3.28
L-Hippocampus	37	15/405	−30 −36 3	3.72
L-Pallidum	NA	17/459	−24 −6 −3	–5.03
**Slow-4 (0.027–0.073 Hz)**				
L-ITG/FG/Hippocampus	20	54/1,458	−36 −3 −42	3.72
L-PreC/PostC	6	16/432	−36 0 30	−3.08

*The results were obtained by two-way repeated-measures ANOVA (*p* < 0.05, *F* > 3.98, uncorrected) and *post hoc* two-sample *t*-test (voxel *p* < 0.05, threshold of *t* > 2.00, uncorrected). BA, Brodmann’s area; MNI, Montreal Neurological Institute; L/R, left/right; ACC/MCC, anterior/middle cingulate cortex; mSFG, medial superior frontal gyrus; ITG, inferior temporal gyrus; FG, fusiform gyrus; PreC/PostC, precentral/postcentral gyrus.*

**TABLE 4:** Correlative analysis of clinical assessments.

Brain regions	Frequency band	LEDD		HY		Duration		UPDRS-III		MMSE	
		*R*	*P*	*r*	*P*	*R*	*P*	*r*	*p*	*R*	*p*
***Brain regions of group analysis of typical band***											
R-ITG/FG/ParaHip	typical	0.02	0.9275	0.25	0.1521	0.20	0.2523	0.14	0.4299	−0.14	
L-FG/ITG	typical	−0.02	0.9212	0.17	0.3281	0.33	0.0561	0.16	0.3714	−0.12	0.4748
L-AG/pMTG	typical	−0.13	0.4487	−0.23	0.1747	−0.24	0.1628	−0.13	0.4642	0.10	0.5722
L-Cuneus/Calcarine	typical	−0.06	0.7213	−0.05	0.7916	−0.17	0.3219	0.04	0.8115	−0.01	0.9646
L-PostC/SMG	typical	−0.17	0.3360	−0.18	0.2977	−0.22	0.2106	−0.38	0.0250	0.20	0.2577
R-Precuneus	typical	−0.14	0.4162	−0.40	0.0178	−0.26	0.1252	−0.42	0.0128	0.22	0.2014
L-Cerebellum_6	typical	−0.28	0.1076	−0.10	0.5557	−0.05	0.7888	−0.18	0.2985	0.24	0.1629
**Brain regions of group effect**											
R-FG/ParaHip	slow-5	0.00	0.9906	0.21	0.2293	0.23	0.1794	0.10	0.5670	−0.29	0.0929
	slow-4	−0.09	0.6055	0.13	0.4529	0.18	0.3111	0.04	0.8077	−0.13	0.4537
R-ITG	slow-5	0.16	0.3474	0.26	0.1271	0.18	0.2904	0.29	0.0909	0.11	0.5186
	slow-4	0.10	0.5668	0.26	0.1268	0.00	0.9888	0.22	0.2057	−0.09	0.5901
L-FG/ITG	slow-5	0.10	0.5664	0.29	0.0867	0.43	0.0095	0.30	0.0804	−0.12	0.4814
	slow-4	−0.09	0.6190	0.10	0.5625	0.23	0.1865	0.08	0.6289	−0.10	0.5763
L-AG/pMTG	slow-5	−0.01	0.9554	−0.15	0.3931	−0.10	0.5735	−0.02	0.9111	0.05	0.7619
	slow-4	−0.17	0.3167	−0.25	0.1474	−0.29	0.0886	−0.13	0.4606	0.07	0.6801
L-Cuneus/Calcarine	slow-5	−0.13	0.4695	0.07	0.6835	−0.07	0.6788	0.04	0.7989	−0.03	0.8598
	slow-4	−0.01	0.9463	−0.10	0.5835	−0.20	0.2602	0.07	0.6738	−0.10	0.5570
L-PostC/SMG	slow-5	−0.20	0.2436	−0.30	0.0756	−0.34	0.0444	−**0.50**	0.0021*	0.21	0.2176
	slow-4	−0.13	0.4435	−0.06	0.7320	−0.13	0.4549	−0.24	0.1587	0.15	0.3746
R-Precuneus	slow-5	−0.11	0.5456	−0.34	0.0482	−0.21	0.2243	−0.37	0.0291	0.35	0.0393
	slow-4	−0.17	0.3333	−0.42	0.0120	−0.28	0.1037	−0.43	0.0109	0.17	0.3312
L-Cerebellum_6	slow-5	−0.28	0.0982	−0.21	0.2263	−0.14	0.4083	−0.24	0.1628	−0.05	0.7851
	slow-4	−0.19	0.2711	−0.01	0.9726	0.12	0.5076	−0.08	0.6558	0.31	0.0716
**Brain regions of *post hoc* analysis**											
L-Caudate/Putamen	slow-5	0.29	0.0920	**0.46**	0.0050*	0.40	0.0187	0.22	0.2084	−0.30	0.0793
L-ACC/mSFG	slow-5	−0.09	0.6086	0.13	0.4627	0.31	0.0689	0.19	0.2733	−0.08	0.6303
L-MCC	slow-5	−0.06	0.7503	0.11	0.5235	0.01	0.9364	0.10	0.5696	−0.09	0.6209
R-ITG	slow-5	0.06	0.7396	0.21	0.2312	0.24	0.1736	0.17	0.3382	−0.09	0.6008
R-ACC	slow-5	−0.26	0.1342	0.10	0.5677	0.10	0.5597	0.25	0.1410	0.05	0.7848
L-Hippocampus	slow-5	0.18	0.3070	0.31	0.0746	**0.45**	0.0061*	0.19	0.2630	−0.01	0.9354
L-Pallidum	slow-5	0.16	0.3707	0.08	0.6543	0.04	0.8197	0.03	0.8511	−0.03	0.8596
L-ITG/FG/Hippocampus	slow-4	0.05	0.7646	0.30	0.0807	0.35	0.0365	0.28	0.1021	−0.07	0.6883
L-PreC/PostC	slow-4	−0.11	0.5126	−0.29	0.0902	−0.33	0.0606	−0.15	0.3963	0.20	0.2493
**Bran regions of analysis within bilateral caudate/putamen**											
L-Caudate/Putamen	slow-5	**0.37**	0.0282*	**0.51**	0.0020*	**0.44**	0.0081*	0.21	0.2179	−0.23	0.1933
	slow-4	**0.40**	0.0177*	**0.54**	0.0008*	**0.44**	0.0080*	0.24	0.1685	−0.06	0.7201

The meaning of the bold values is equal to “*” meaning signiffcant results after correction by false discovery rate (FDR) with p < 0.05.

**FIGURE 5:**
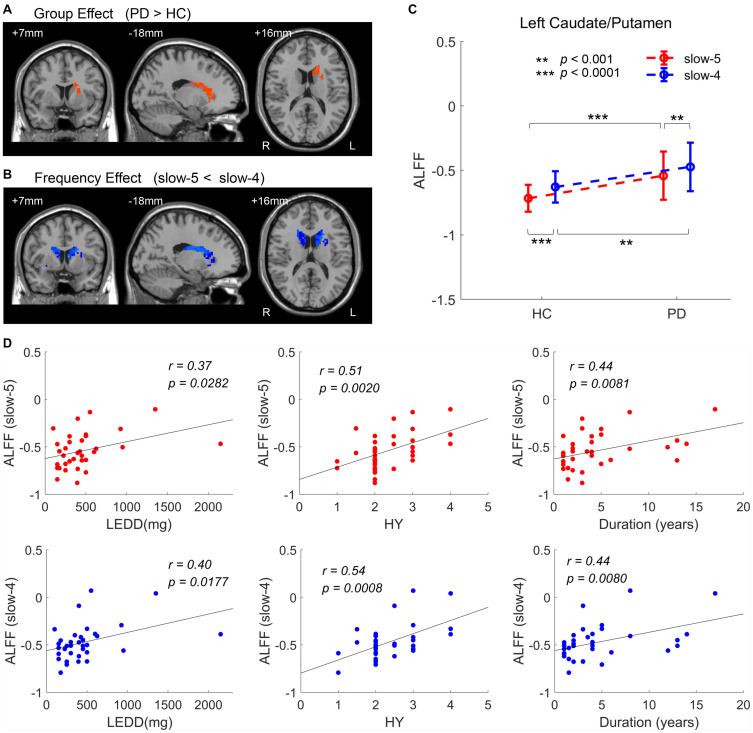
ANOVA within the bilateral caudate/putamen. **(A)** Group effect [GRF corrected, voxel *p* < 0.05, cluster *p* < 0.05, threshold of *t* > 2.00, and cluster size >69 voxels (1,863 mm^3^)]. **(B)** Frequency effect [GRF corrected, voxel *p* < 0.05, cluster *p* < 0.05, threshold of *t* > 2.00, and cluster size >50 voxels (1,350 mm^3^)]. **(C)** ALFF of two groups within the significant region of group effect, the left caudate/putamen. **(D)** Clinical correlations within the significant region of group effect (the left caudate/putamen) (FDR corrected, *p* < 0.05). L/R, left/right; LEDD, levodopa equivalent daily dose; HY, Hoehn and Yahr scale. **p < 0.001, ***p < 0.0001.

Results of analysis within the bilateral caudate/putamen were shown in Figure 5. Statistical maps were corrected by GRF with voxel *p* < 0.05 (*t* > 2.00) and cluster *p* < 0.05. Analysis of group effect showed that PD had higher ALFF in the left caudate/putamen (113 voxels, 3,051 mm^3^) (Figure 5A). Analysis of frequency band effect demonstrated that ALFF of slow-4 was observed to be higher than slow-5 in the bilateral caudate/putamen (516 voxels, 13,932 mm^3^) (Figure 5B). No significant results were found in the interaction analysis under GRF correction with voxel *p* < 0.05. Additionally, for both the two groups and two frequency bands, ALFF signals were extracted from the left caudate/putamen within which showed a significant group effect, respectively. In addition to the group effect, significantly higher ALFF of slow-4 than that of slow-5 was observed in the two groups in the left caudate/putamen (Figure 5C). The ALFF of PD showing a group effect in Figure 5A was positively correlated with LEDD, HY stage, and disease duration in both slow-5 and slow-4 bands (Figure 5D and Table 4). All results of correlation between the abnormal brain regions with the clinical assessments are shown in Table 4. Besides the significant correlations mentioned previously, the decreased ALFF in the left PostC/SMG showing a group effect (Table 2 and Figure 2) was negatively correlated with UPDRS-III score (*p* < 0.05, FDR corrected).

## Discussion

Using the ALFF method, our study found that compared with the HC group, PD patients exhibited significantly abnormal ALFF in widespread brain regions. Moreover, the abnormal ALFF in some brain areas was different in specific frequency bands. Our results suggest that PD is associated with widespread abnormal brain activities and that the abnormalities are associated with specific frequency bands.

### ALFF Differences Between the PD and HC Groups

In this study, we observed increased ALFF in PD patients predominantly in several parieto-temporo-occipital regions, such as ITG/FG and AG/pMTG, which is thought to be crucial to visual and semantic functions (Kravitz et al., 2013; Davey et al., 2015). A study of PD with hallucination (Yao et al., 2015) in the “on” state found that PD patients with or without hallucination exhibited similar increased ALFF in the medial temporal lobe and temporo-parietal gyrus compared with HC, which was consistent with our results. Abnormal regional cerebral blood flow has been observed in the right FG, MTG, and superior temple gyrus in PD patients with visual hallucinations in the on-medication condition (Oishi et al., 2005). However, increased ALFF in some temporal regions (e.g., the left superior temporal gyrus, left ITG, and right FG) was also observed in PD patients in another study, but the medication condition was unclear (Zhang et al., 2015). Although the exact mechanisms underlying these findings are not clear, it is tempting to hypothesize that increased ALFF in these cortices may imply greater neuronal activities at rest and possibly intend to maintain the function or may be related to the therapy of dopamine drugs.

Lower ALFF in the left cerebellum was discovered in our study compared with controls, which was consistent with several R-fMRI studies (Skidmore et al., 2013; Zhang et al., 2013). However, much evidence has demonstrated that hyperactivation in the cerebellum exists in PD patients who withdrew from medication for at least 12 h (Yu et al., 2007; Wu et al., 2010). Generally, pathological impairments should be more severe as disease progresses because dopaminergic degeneration develops gradually (Jankovic, 2005). The major roles of the cerebellum in PD include pathological and compensatory effects. The compensatory effect exists and strengthens at a relatively early stage to maintain relatively motor and non-motor functions. However, as the pathological effects progress gradually, the compensatory effect may diminish or eventually fail at the advanced stage. The decreased ALFF in the cerebellum might be related to dopaminergic medication that weakens the compensatory effect or to serious pathological progression that does not respond to dopaminergic medication.

Similarly, a previously reported decrease in ALFF in the right precuneus and left PostC/SMG (Cavanna and Trimble, 2006; Juenger et al., 2011) that is closely related to cognitive and somatosensory functions was also observed in this study. A study of non-demented PD in the “ON” state showed that cerebral blood flow (CBF) in the precuneus was significantly reduced (Syrimi et al., 2017). Furthermore, precuneus hypoperfusion was identified in a perfusion study of PD with mild cognitive impairment, and reduced precuneus function connectivity in the striatum was further detected in participants’ off-medication state (Jia et al., 2018). Diminished activation within the somatosensory cortex has been demonstrated in PD patients; furthermore, dopaminergic medications did not alter the deficient activation of the primary somatosensory cortex (Nelson et al., 2018). It seems that decreased ALFF in the precuneus and somatosensory cortex might be induced by pathological changes in dopaminergic degeneration and was uneasily corrected by dopaminergic medication.

Numerous studies have reported abnormal spontaneous brain activities in some regions important for motor control. A meta-analysis (Pan et al., 2017) showed that decreased ALFFs in the bilateral supplementary motor areas and left premotor cortex were the most consistent and replicable findings related to motor function in PD patients, which were not found in our study. Moreover, it has been proven that spontaneous brain activity deficits in the left premotor cortex, inferior frontal gyrus, and supplementary motor area can been restored after treatment and were no longer different from those in controls (Possin et al., 2013). The normalization of ALFF in the motor areas in this study is likely related to dopaminergic medication.

### ALFF Differences Between the Frequency Bands

In this study, the main effect of the frequency band obviously showed that greater ALFF in the slow-5 band was identified in widespread cortical regions, whereas greater ALFF in the slow-4 band was identified mainly in the temporal cortex and subcortical regions. These results are consistent with the frequency effect analysis in other studies (Han et al., 2011; Hou et al., 2014; Liu et al., 2014). The slow-5 band (0.010–0.027 Hz), which has more power, is associated with the integration of large-scale neural networks and long-distance connectivity and localized mainly to the prefrontal, parietal, and occipital cortex, whereas the slow-4 band (0.027–0.073 Hz), which has less power, is linked with more local neural activity and short connections and localized more within the temporal cortex and subcortical structures, such as the thalamus and basal ganglia (Zuo et al., 2010; Baria et al., 2011). The human brain is a complex biological system generating a multitude of oscillatory waves that cover a wide range of frequencies, and the signals within different frequency bands may be derived from distinct oscillators with specific properties and physiological functions (Buzsaki and Draguhn, 2004). However, the origins, relationships, and specific physiological functions of different frequency bands have not be completely clarified to date. Because BOLD oscillations have been proven to directly associate with electroencephalographic signals (He et al., 2008), future work can identify the neurophysiological basis of specific frequency bands by combining R-fMRI with electroencephalographic recordings.

### Frequency-Dependent Changes in ALFF in PD Patients

The abnormal ALFF in some brain areas of PD patients (Table 3 and Figure 4B) was detected different in specific frequency bands (uncorrected). Especially, the increased ALFF in the left caudate/putamen was more prominent in the slow-5 band compared with the slow-4 band in our on-medication research. Decreased ALFF in the caudate was observed in the slow-4 band in a study performed in the off-medication state (Hou et al., 2014). Studies related to the specific frequency band of PD are rare, and no exactly consistent and generally accepted results have been detected to date. One brain region may present multiple oscillations belonging to different frequency bands. The inconsistent changes of the brain region within the same frequency band may result from various reasons, such as the heterogeneity in samples and the confounding effect (e.g., ON medication or OFF medication) on results between group comparisons. Research on dopaminergic drug effects has proven that the functional connectivity of the caudate nucleus can be modulated specifically by dopaminergic drugs (Honey et al., 2003). Several previous studies showed that levodopa administration can normalize the activity pattern in PD, including increasing neural activity in the striatum (including the caudate and putamen) (Kraft et al., 2009; Wu et al., 2009). It is likely that in PD, hypoactivation in the striatum as the consequence of disorder progression-related pathological neural changes may be more sensitively explored in the slow-4 band, whereas hyperactivation in the striatum as the neural alterations related to dopaminergic drugs may be more sensitively detected in the slow-5 band. The differences of PD-related activity between frequency bands suggested that the abnormalities in intrinsic brain activity in PD patients were associated with specific frequency bands. The properly chosen frequency band can be helpful to more sensitively explore PD-related neural changes.

Although ANOVA was performed within the gray matter mask, the cluster of the left caudate/putamen seemingly has a low percentage, extending to the white matter of the underlying mask, which was difficult to calculate. Besides pathological changes of neural bodies mainly located in the gray matter or gray matter nuclei, studies have demonstrated that damage to the projecting axons of substantia nigral neurons also happened in PD (Tagliaferro et al., 2015). Most previous studies of low-frequency fluctuation were focused on the gray matter for its postsynaptic potential, whereas Ji et al. (2017) considered that low-frequency BOLD fluctuations in the white matter were also significant and can be used to estimate the dynamic functioning of fiber tracts. Ji et al. (2019) have found decreased structural–functional coupling in the left corticospinal tract, and this tract displayed abnormally increased functional connectivity within the left PostC and left putamen in PD patients. More functional investigation of the white matter may be helpful to understand the pathophysiology of PD.

### Correlation Analysis

The left caudate/putamen detected in the slow-5 band as a result from ANOVA and *post hoc* analysis was positively correlated with the HY stage. Moreover, the left caudate/putamen resulting from the group effect of ANOVA in the template of the bilateral caudate/putamen was positively correlated with LEDD, HY stage, and disease duration. These accordant positive correlations indicate that as the disease continues, the course progresses, and the LEDD increases, the neural activity in the left caudate/putamen becomes more intensive. PD is a progressive neurodegenerative disorder caused by the gradual degeneration of dopaminergic nigrostriatal neurons. As the disease continues and the course progresses, the LEDD gradually increases. The striatum and thalamus were thought to be the regions with the most prominent response to levodopa within the cortico-subcortical motor circuit (Kraft et al., 2009). ALFF in the caudate nucleus has also been detected to be positively correlated with the dose of levodopa in an R-fMRI study (Hou et al., 2014). In the on-medication state, it can be easily understood that the positive correlation was mainly attributed to dopaminergic medication in our study.

The negative correlation of the left PostC/SMG with the UPDRS-III score found in our study means that the brain activity of the left PostC/SMG gradually decreased as motor dysfunction worsened. The PostC is a cortical center for somatosensory processing, and abnormalities in this region may be responsible for the disrupted perception of tactile, painful, thermal, and proprioceptive inputs in PD. More importantly, it may superimpose the so-called abnormal background noise on motor function, hampering sensorimotor integration and ultimately resulting in clinical motor symptoms (Conte et al., 2013). Indeed, different from the past understanding, motor symptoms in PD are not purely motor problems now; it has been proven to be associated with types of cognitive impairments (Wojtala et al., 2019). PD subjects have demonstrated diminished activation within the somatosensory cortex, and it was insensitive to dopaminergic medications (Nelson et al., 2018). A meta-analysis (Ji et al., 2018) found that abnormalities of the PostC were unaffected by medication as well. Overall, the abnormal neural activity in the PostC of PD patients decreases as symptoms progress and has the potential to be used as a biomarker for predicting the severity of PD.

Although the findings of our study are encouraging, several limitations must be addressed. First, the present study was limited by a relatively small sample size. Therefore, the results of the current study should be replicated with a larger sample. Second, although the age was included as a covariate to reduce its effect, age was not well matched between the PD and HC groups because the *p*-value of age is actually not large enough. Third, we were unable to distinguish all the effects of medication on neural activity from progression-related pathological neural change. With further available comparison studies, investigation of PD related to “on” or “off” medication states will be more important.

## Conclusion

In the present study, we found that PD patients exhibited increased ALFF in many parieto-temporo-occipital regions along with decreased ALFF in the cerebellum and some parietal cortex in a typical band. In particular, we found that the abnormalities of activity in some brain regions, such as the left caudate/putamen, in PD patients are different in specific frequency bands (uncorrected). Our findings suggest that the use of specific frequency bands will be helpful in detecting the neural changes in PD, which should be considered in future works.

## Data Availability Statement

The raw data supporting the conclusions of this article will be made available by the authors, without undue reservation.

## Ethics Statement

The studies involving human participants were reviewed and approved by Institutional Review Board (IRB) of Guangzhou First People’s Hospital. The patients/participants provided their written informed consent to participate in this study.

## Author Contributions

ZW wrote the manuscript. ZW, YaL, and XW conceived of the idea and performed the literature review. YuL, ML, and XR performed the data analysis. YuL, EL, and GZ contributed to the data collection. All authors interpreted the results, reviewed the manuscript, and joined the discussion of the manuscript.

## Conflict of Interest

The authors declare that the research was conducted in the absence of any commercial or financial relationships that could be construed as a potential conflict of interest.

## Footnotes

1 http://www.rfmri.org/dpabi

2 http://www.fil.ion.ucl.ac.uk/spm
